# Taxonomy of anaerobic digestion microbiome reveals biases associated with the applied high throughput sequencing strategies

**DOI:** 10.1038/s41598-018-20414-0

**Published:** 2018-01-31

**Authors:** Stefano Campanaro, Laura Treu, Panagiotis G. Kougias, Xinyu Zhu, Irini Angelidaki

**Affiliations:** 1 Department of Biology, University of Padova, Via U. Bassi 58/b, 35121 Padova, Italy; 2 Department of Environmental Engineering, Technical University of Denmark, 2800 Kgs Lyngby, Denmark

## Abstract

In the past few years, many studies investigated the anaerobic digestion microbiome by means of 16S rRNA amplicon sequencing. Results obtained from these studies were compared to each other without taking into consideration the followed procedure for amplicons preparation and data analysis. This negligence was mainly due to the lack of knowledge regarding the biases influencing specific steps of the microbiome investigation process. In the present study, the main technical aspects of the 16S rRNA analysis were checked giving special attention to the approach used for high throughput sequencing. More specifically, the microbial compositions of three laboratory scale biogas reactors were analyzed before and after addition of sodium oleate by sequencing the microbiome with three different approaches: 16S rRNA amplicon sequencing, shotgun DNA and shotgun RNA. This comparative analysis revealed that, in amplicon sequencing, abundance of some taxa (*Euryarchaeota* and *Spirochaetes*) was biased by the inefficiency of universal primers to hybridize all the templates. Reliability of the results obtained was also influenced by the number of hypervariable regions under investigation. Finally, amplicon sequencing and shotgun DNA underestimated the *Methanoculleus* genus, probably due to the low 16S rRNA gene copy number encoded in this taxon.

## Introduction

A fundamental step to uncover microbial community structure and dynamics is the taxonomic and phylogenetic classification of DNA sequences. More than 30 years ago these studies were revolutionized by the introduction of molecular techniques to investigate the small-subunit rRNA sequence (16S rRNA)^1^. Subsequently, the combination of PCR and high-throughput sequencing made these studies extremely popular among the scientific community^2–4^. Four fundamental factors contributed to the success of these approaches: (1) PCR amplification which allows the generation of amplicons from small amount of starting material^5^, (2) high throughput sequencing and molecular barcoding supports parallel analysis of numerous samples, (3) availability of simple bioinformatics tools which simplify the analyses^6,7^ and (4) the growing size of 16S rRNA gene databases^8,9^. Due to these technical advances, in the last few years the number of scientific publications based on 16S rRNA amplicon sequencing underwent an impressive increase.

Despite this success, accuracy of PCR-based approaches is limited by different factors, such as biases in the range of species targeted by universal primers and the generation of chimeras during amplification^10–12^. Another important factor that should be considered is the length of the sequences used for taxonomical investigation; in fact, short reads generated by high-throughput platforms may represent a limitation for taxonomic assignment. Despite this, it was reported that 16S rRNA reads as short as 100 bp allow an accurate characterization of a microbial community^13^. Finally, utilization of different sets of universal primers to measure abundance of Bacteria and Archaea can prevent a thorough comparison of the Operational Taxonomic Units (OTUs) belonging to these two kingdoms.

In the past few years some studies compared the results obtained using 16S rRNA amplicon sequencing with other approaches in order to reveal biases and limitations. The vast majority of these projects analyzed “real” microbial communities isolated from diverse environments such as soil^12^, water lake^14^, marine samples^15^, gut samples^16^, spleen^17^ and anammox bioreactors^18^. Only a few of these studies focused the investigation on mock (controlled synthetic) communities^12,19^. To our knowledge, there are not investigations focusing on the anaerobic digestion (AD) microbiome. Additionally, the projects mentioned above were focused on two approaches such as amplicons and shotgun DNA sequencing (metagenomics)^12,14,15^ or, alternatively, amplicons and RNA sequencing (metatranscriptomics)^16–18^, but a comprehensive comparative evaluation is still lacking.

Complex microbial interactions are occurring in the AD process and a balanced microbial community composition is crucial for a well performing process. Therefore, the relevant ecological role of the AD microbiome has led the scientific community to deserve particular attention to this system^20,21^. Additionally, operational parameters have a significant impact on the microbial compositions and therefore taxonomic investigation is required to elucidate these correlations. In the past few years, a plethora of studies investigated this microbiome using different molecular approaches such as DGGE^22,23^, 16S rRNA amplicon sequencing^24–27^, metagenomics^28,29^, metatranscriptomics^30–32^ and genome-centric metagenomics^33–35^. Most of these studies compared taxonomic results obtained using different methods, however, it should be noticed that limitations and biases associated with different approaches, can lead to misleading interpretations. In the present study we performed a taxonomic investigation of the AD microbiome present in laboratory-scale manure-based reactors characterized by different concentrations of unsaturated long chain fatty acids. In order to identify strengths and limitations associated with different approaches, sequences were generated using as template 16S rRNA amplicons, genomic DNA and total RNA collected from the same samples. Numerous analyses were performed to specifically identify the origin of biases, including for example a comparison between databases used for reference-based taxonomic assignment. Additionally, this study led to the identification of biases associated with the use of universal primers in the PCR amplification step.

## Results and Discussion

### Experimental setup

The microbial communities under investigation were grown in three laboratory scale Continuous Stirred Tank Reactors (CSTR) operated at thermophilic conditions (54 ± 1 °C) and fed with cattle manure. Samples were collected twice from each reactor, the first sampling was performed when the influent feedstock was composed only by cattle manure, the second sampling when the feedstock was added with Na-oleate at a concentration of 12 g/L. Despite addition of long chain fatty acids has a relevant effect on microbial composition^36–39^, the present study does not focused on the interpretation of biological data, but on investigation of potential biases determined by different high-throughput sequencing approaches on taxonomic results obtained. For this reason, biological results are not thoroughly discussed in the paper. To identify these biases it is important to reduce as much as possible the sources of variability that could rise during samples collection, such as lysis of bacterial cells and extraction of nucleic acids. To achieve this goal, both DNA and RNA were extracted using the same kit and protocols used for Illumina sequencing were very similar for all the samples (see Materials and Methods for details).

**Figure 1:**
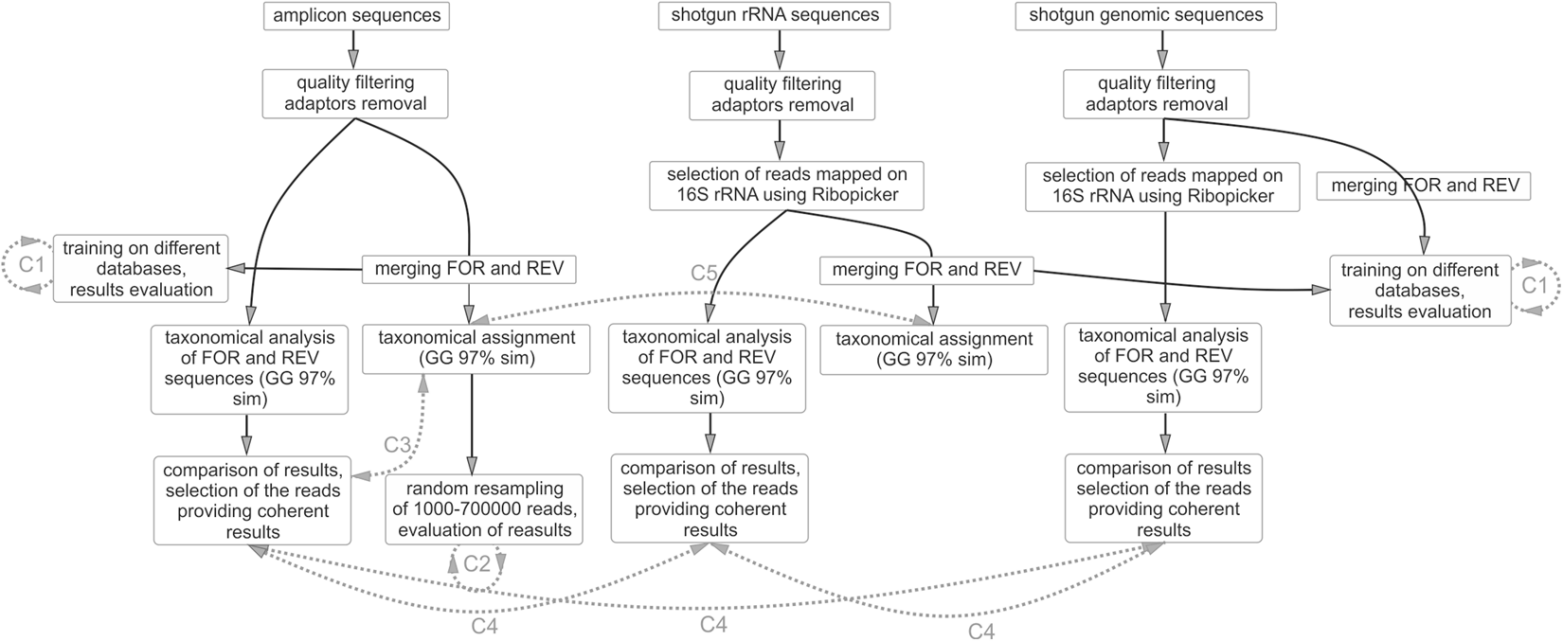
Outline of data analysis process. Gray boxes and black arrows represent the analysis workflow, gray dotted lines represent comparisons between different approaches used for data analysis. (C1) Influence of the database used to train the Bayesian classifier; (C2) influence of the number of reads on taxonomic results; (C3) influence of paired-end reads merging on the taxonomy; (C4) comparison between different 16S rRNA sequencing approaches (with independent taxonomic analysis of forward and reverse paired-ends); (C5) comparison between different 16S rRNA sequencing approaches (using merged paired-ends).

Regarding bioinformatics analysis, the procedure is depicted in Fig. 1 and a detailed description is reported in Materials and Methods section. Four main bioinformatics investigations were performed (Fig. 1). (1) Evaluation of the influence determined by the database used for training the Bayesian classifier (RDP, Greengenes and SILVA) (Fig. 1, C1)^8,9^. (2) Calculation of the minimum number of sequences needed in order to obtain a “solid and reliable taxonomic” result (Fig. 1, C2). (3) Influence on taxonomic assignment of the “Forward” and “Reverse” reads merging step (Fig. 1, C3). (4) Influence of the sequencing method used (amplicon sequencing, shotgun RNA sequencing and shotgun DNA sequencing) (Fig. 1, C4 and C5). The latter bioinformatics check was performed both without preliminary merging forward and reverse read pairs (C4) and also after the paired-end merging step (C5). The loss of reads due to the merging step led to a marked reduction of the data available for the comparison; for this reason the step “C5” was performed only on the sequencing methods which provided the highest number of sequences mapped on 16S rRNA gene: amplicon sequencing and shotgun RNA sequencing.

### Influence of the training set on taxonomic results

**Figure 2:**
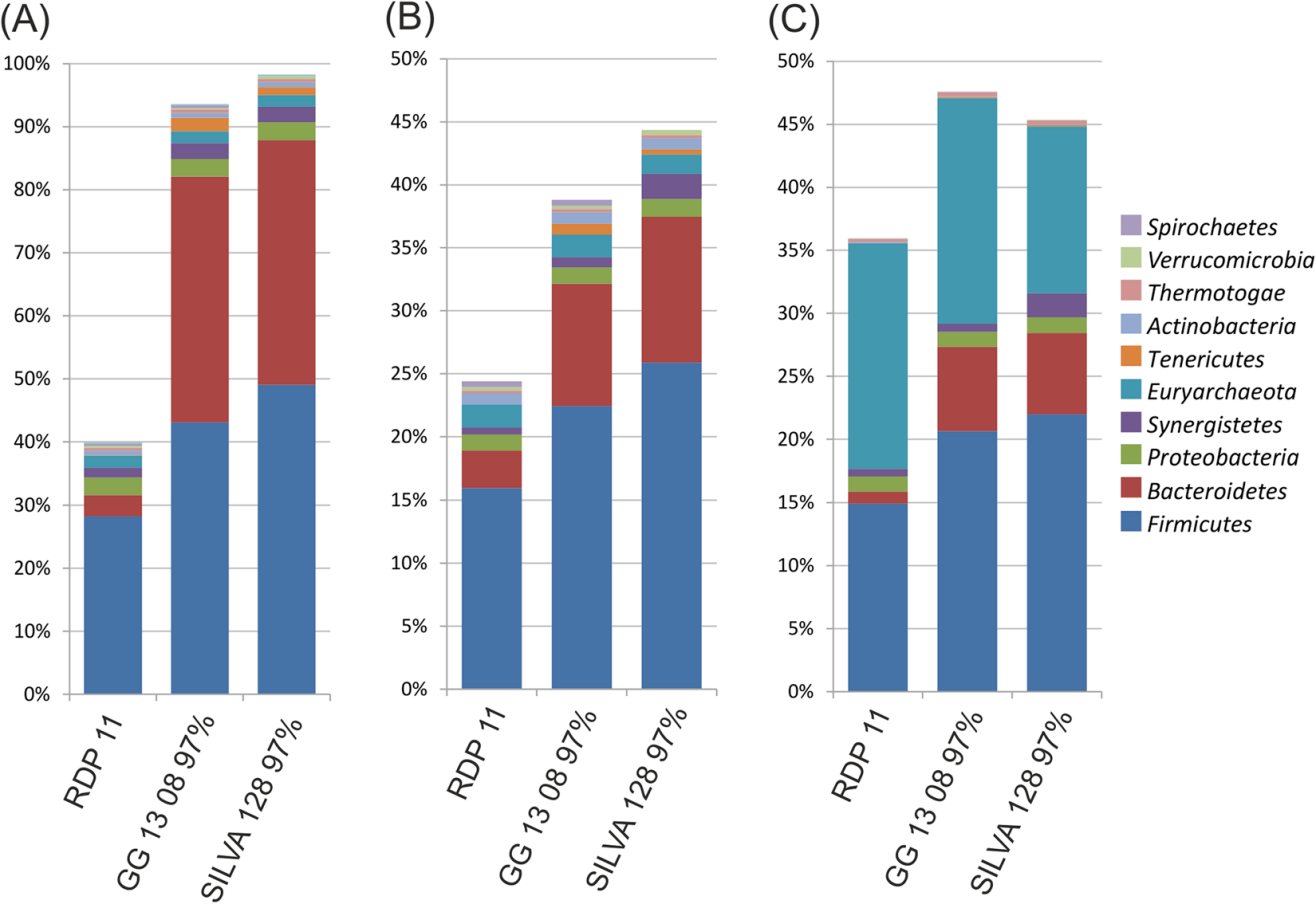
Relative abundance of the ten major phyla identified in CSTR01 sample. Results were obtained from (**A**) ~712,000 16S rRNA amplicons, (**B**) ~34,000 shotgun DNA and (**C**) ~976,000 shotgun RNA sequences aligned to the 16S rRNA gene. Numbers refer to the reads obtained after paired-end merging. Results were obtained after training the na*i*ve Bayesian classifier on different databases (RDP release 11, Greengenes 13 08 and SILVA release 128).

It was previously reported that different training sets can have a remarkable influence on the number of reads that the Bayesian RDP Classifier can assign to the taxonomy and on the abundance calculated for different taxa^40^. In the same study it was also reported that number of unassigned reads is lower for well-characterized microbial communities (e.g. the human gut microbiome), while it is more problematic when many unknown species are dominant. To test the influence of the training set on the taxonomic investigation of the AD microbiome, RDP classifier was trained using three different databases, RDP, Greengenes and SILVA. After training, a taxonomic assignment was performed and results obtained at phylum level for sample CSTR01a were reported in Fig. 2 (Supplementary Table S1). These results evidenced that at phylum level the highest number of taxonomic assignments were obtained using SILVA and the lowest were obtained using RDP (Fig. 2). The limitations associated with the use of RDP database in the taxonomic investigation of the AD microbiome was also reported in previous studies^40^. Unfortunately, a more detailed analysis performed at taxonomic level lower than phylum revealed that ∼160,000 sequences were not assigned in a reliable way, but were allocated to “uncultured taxa”. Due to the difficulty in filtering out these “fake” assignments and in calculating abundance at low taxonomic levels, results obtained using Greengenes was selected for all the subsequent analyses.

Biological results obtained are in agreement with previous data, with a vast majority of sequences assigned to *Firmicutes*, *Bacteroidetes* and *Proteobacteria*, the three main phyla characterizing the AD microbiome (Fig. 2)^29,41–44^. Interestingly, using RDP release 11, the fraction of sequences assigned to *Bacteroidetes* and *Tenericutes* is extremely low, evidencing that results are strongly biased by the training set (Fig. 2). Analysis of the shotgun RNA reads gave strongly different results in comparison to the other two methods (Fig. 2C) and this can be due to differences in transcriptional activity of some microbes or to the 16S rRNA gene copy number, as described more in detail in section “Comparison between results obtained using different sequencingmethods”. By considering the reads aligned to the 16S rRNA gene, it is evident that for amplicon sequencing the fraction of those assigned to specific phyla can be higher than 90% (Fig. 2A). For shotgun DNA and shotgun RNA this value is lower than 50% (Fig. 2B and C). This marked difference is due to the random distribution of the shotgun reads and, more specifically, to those aligned to the conserved regions of the 16S rRNA gene. These specific regions are highly conserved among different taxa and useless in taxonomic analysis. Obviously, in the 16S rRNA amplicon sequencing all the reads are localized on hypervariable regions and this represents an advantage because all of them are taxonomically informative.

### Minimum number of sequences required for a reliable taxonomic investigation

**Figure 3:**
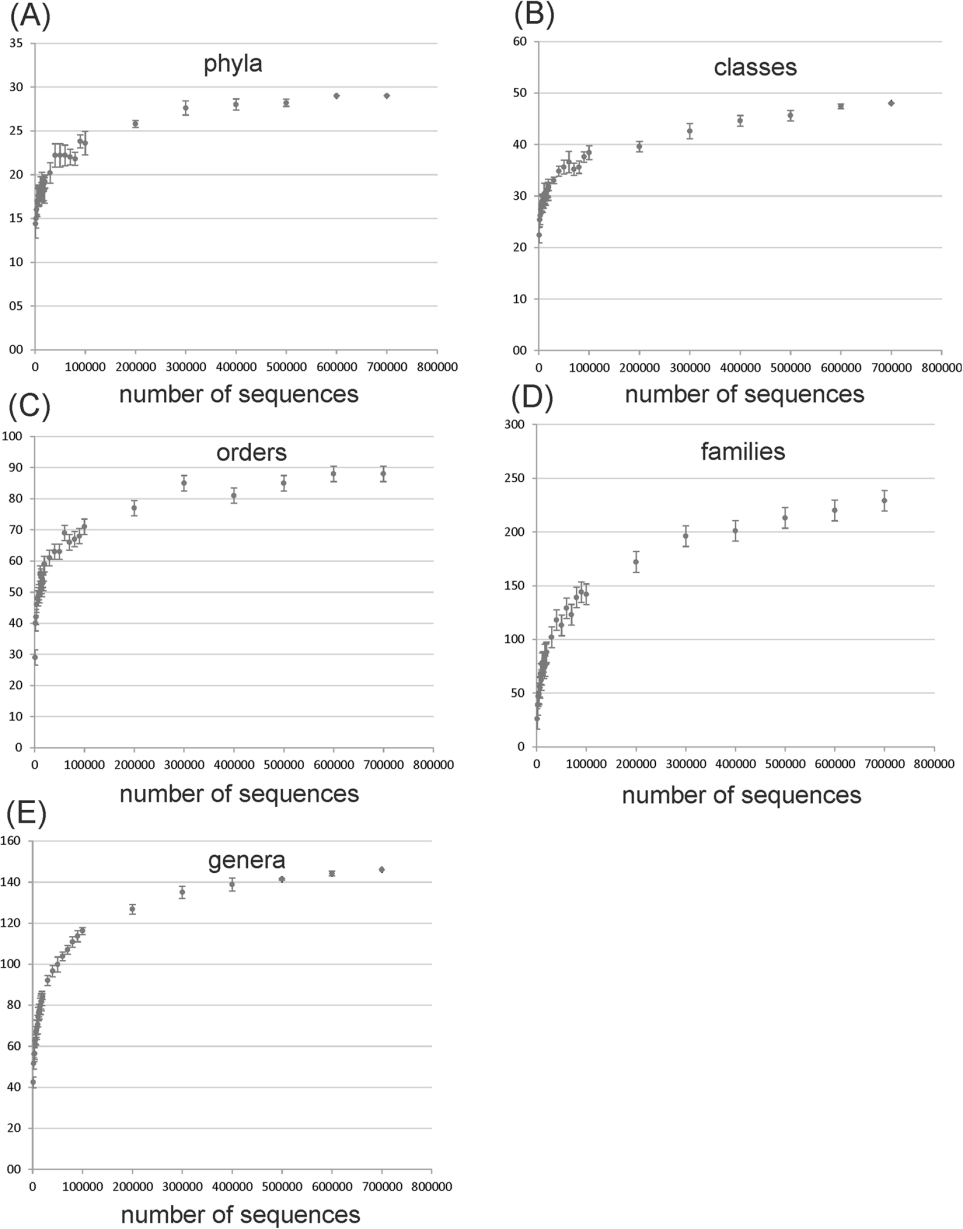
Number of taxa identified with an increasing number of reads. Reads from amplicons (merged paired-end) were random resampled starting from a minimum number of 1000 sequences up to 700,000 sequences. After taxonomic analysis the number of phyla, orders, families, classes and genera was calculated and reported in y axes.

The 16S rRNA gene(s) represent less than 1% of the entire genome and for this reason the fraction of shotgun DNA reads assigned to this gene is low^15^ (see also Methods). This problem can prevent the investigation of the rare taxa, thus, a rarefaction approach was used in order to verify the minimum number of reads needed for a reliable taxonomic analysis in samples obtained from biogas reactors. The random sampling was performed on 16S rRNA amplicons starting from 1000 reads, increasing stepwise the number up to 700,000 and repeating five times the taxonomic analysis for each step (Fig. 3). Despite results reported in Fig. 3 indicates that more than 200,000 reads were needed to reach a *plateau* in the number of taxonomic groups, the most abundant ones were already identified with a lower number of sequences. In particular, 29% of genera, 49% of families, 56% of orders, 60% of classes, and 59% of phyla were identified with 10,000 sequences. These values increased up to 62%, 81%, 81%, 79% and 76% using 100,000 sequences. It should be noticed that all the taxonomic groups with abundance higher than 0.1% can be identified (with 10 or more sequences) using as low as 10,000 reads. This finding indicates that, using the shotgun DNA approach and a number of sequences around 60–70 thousand (as in the present study), the analysis can be deep enough to cover most of the taxonomic groups. This result can be considered as a reference to determine the number of 16S rRNA reads needed to analyze the AD microbiome.

### Comparison between results obtained using different sequencing methods

The main topic of the present study is the investigation of biases in taxonomic results associated with the three sequencing approaches used. It was possible to identify both differences determined by PCR amplification biases (16S rRNA amplicons), and those determined by the expression level of rRNA genes (shotgun RNA sequences).

**Figure 4:**
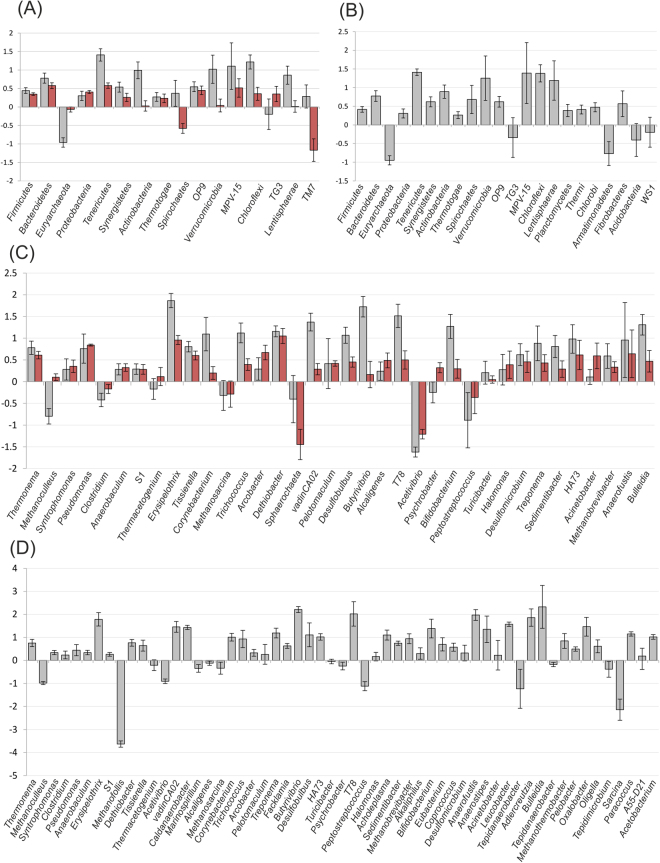
Comparison between abundance of different taxa determined using three sequencing approaches. Results are reported as average of the results determined in the six samples examined (CSTR01a-03a; CSTR01b-03b). The log_2_ ratios of the abundances calculated comparing two different approaches are reported in y axes. Grey bars represent comparison between amplicon sequencing and shotgun RNA, red bars represent the comparison between amplicon sequencing and shotgun DNA. Taxa having higher abundance in amplicons in comparison to shotgun RNA sequencing are reported as gray bars with positive values, those having higher values in amplicons in comparison to shotgun DNA are reported as red bars with positive values. (**A**) Comparison at phylum level between amplicons, shotgun RNA and shotgun DNA (for and rev sequences analyzed separately); (**B**) comparison at phylum level between amplicons and shotgun RNA; (**C**) the same comparison reported in (**A**) at genus level; (**D**) the same as reported in (**B**) at genus level. In (**B**) and (**D**) analysis was performed on 700,000 sequences obtained after merging for and rev paired-ends.

Since the number of sequences mapped on the 16S rRNA gene is strongly variable among the three approaches used, we performed an initial investigation by subsampling, selecting randomly 60,000 reads for each sample. This number was chosen considering the less numerous sample of the random DNA sequencing (Supplementary Table S2). From this analysis it was found that two main phyla were strongly under-represented in the amplicon analysis in comparison to random shotgun DNA sequencing (Fig. 4A, red bars): *Spirochaetes* and Candidate Division TM7 (Candidatus *Saccharibacteria*). On contrary, *Euryarchaeota* phylum was highly-represented only in random RNA sequencing, suggesting a very high transcriptional activity (Fig. 4A). The *Methanoculleus* genus was the main responsible for this result (Fig. 4C). This peculiar characteristic of methanogenic Archaea was previously reported and associated to the remarkable transcriptional activity of the genes involved in methanogenesis^30,31^. A possible alternative explanation for the results obtained for *Euryarchaeota* (*Methanoculleus*) could be the presence of a low number of 16S rRNA genes encoded in the genome. This is a peculiar characteristic of some taxa which can result in an underestimation of the abundance determined with amplicons and shotgun DNA^45^. Differently from this, the gene copy number has a low impact on shotgun RNA which is influenced only by the expression level. In order to investigate the influence of gene copy number, it was calculated the average number of 16S rRNA genes on each taxonomic group^46^. Results indicated that, among taxa reported in Fig. 4, those with the lower rRNA gene copy number were *Chloroflexi*, *Tenericutes*, *Spirochaetes*, *Verrucomicrobia*, *Thermotogae* and *Euryarchaeota*. Among these, only *Euryarchaeota* have a markedly higher abundance value estimated with the shotgun RNA method, suggesting that gene copy number can influence the results obtained with shotgun DNA and amplicons, but it is not the only determinant. An independent analysis was performed determining abundance levels for all the taxa with MetaPhlAn 2 software^47^, which can align the shotgun DNA sequences on unique clade-specific marker genes other than the 16S rRNA. Interestingly, results obtained with MetaPhlAn 2 correlated well with those obtained with shotgun RNA, confirming that there is a substantial underestimation of *Euryarchaeota* determined by the utilization of the 16S rRNA marker gene both in amplicon and shotgun DNA sequencing (Supplementary Table S3). Since this bias is absent in the “shotgun RNA” based approach, it is probably due to the 16S rRNA gene copy number, but it remains unclear why it does not influence other phyla characterized by a low 16S rRNA copy number.

In amplicon sequencing a substantially lower abundance of genera, such as *Methanosarcina*, *Sphaerochaeta*, *Acetivibrio* and *Peptostreptococcus*,was found (Fig. 4C). This result was confirmed both by shotgun RNA and DNA sequencing.

A second analysis was performed only on the two sequencing approaches providing the highest number of 16S rRNA reads (16S amplicon sequencing and shotgun RNA sequencing). In this comparison 700,000 sequences were collected per each sample (Fig. 4B and D) (Supplementary Table S4). This investigation was performed using the sequences obtained after paired-end merging. It should be noted that in the present study, independent analysis of forward and reverse reads provided very similar results in comparison to the use of merged paired-ends, as reported in Supplementary Dataset S1. Moreover, the threshold of the Bayesian classifier was decreased to 0.5 to verify also the presence of sequences belonging to taxa more difficult to identify. Despite the modifications introduced in the parameters, results obtained substantially confirmed the previous ones indicating that analysis is solid. An interesting difference was found regarding *Armatimonadetes* phylum, which was identified at higher abundance in the shotgun RNA sequencing.

### Comparison of results obtained investigating different hypervariable regions

**Figure 5:**
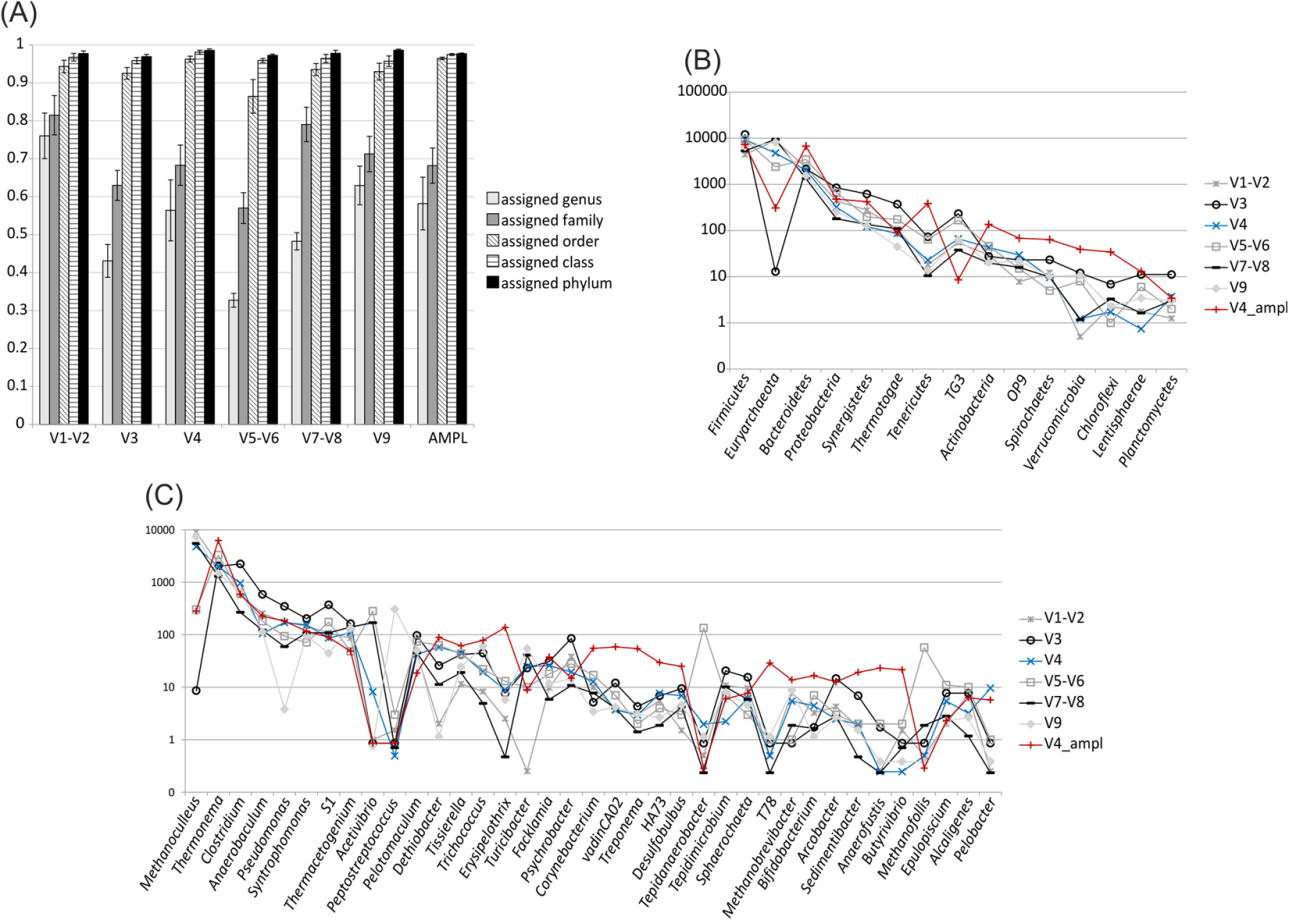
Abundance of different taxa calculated considering shotgun rRNA sequences assigned to different hypervariable regions. (**A**) Fraction of sequences assigned to different taxonomic levels and normalized considering the total number of sequences assigned to each hypervariable region. Number of reads assigned to different phyla (**B**) and genera (**C**) calculated considering reads assigned to different hypervariable regions. Notice the logarithmic scale on y axes (number of sequences) in (**B**) and (**C**). In (**A**) all the six samples are reported and variability is represented as standard deviation on each bar, in (**C**) results are reported for sample CSTR01a.

As reported in section 2.4, different sequencing approaches revealed discrepancies in abundance of specific taxa. This can be determined by two main effects: (1) different distribution of the reads on the 16S rRNA gene, and (2) biases in amplification of universal primers used for PCR. Regarding the first effect, it should be evidenced that amplicon sequencing targets specifically the V4 region, while shotgun sequencing allows investigation of multiple hypervariable regions. It was previously reported that different hypervariable regions can provide different results in the taxonomic assignment^48^. To determine the contribution of specific hypervariable regions to the taxonomic assignment, sequences derived from shotgun RNA were classified in six main classes according to their position on the 16S rRNA gene sequence (Fig. 5) (Supplementary Table S5). Some regions (V1-V2, V5-V6 and V7-V8) were not considered singularly, but were investigated as couples because they were shorter than the average length of the shotgun sequences. Analysis was performed only on shotgun RNA because the number of reads was very high and this allowed a reliable analysis even after subsampling the reads on hypervariable regions. On contrary, the low number of sequences obtained for shotgun DNA made this analysis unreliable.

An evaluation of the number of sequences assigned to the taxonomy revealed that the best result was obtained using those aligned to regions V1 and V2 (Fig. 5A). This was more evident at low taxonomic levels (e.g. genus and family). Similar evidences were found by previous analyses performed on activated sludge^48^ suggesting that V1-V2 are good target regions for taxonomic analysis. At genus level the percentage of reads taxonomic assigned varied from 33% on V5-V6 regions to 76% on V1-V2 regions. As expected results obtained considering only sequences positioned on the V4 region was similar to those obtained using amplicons (Fig. 5A). These findings indicate that the hypervariable region used for classification is one of the main determinants for the discrepancies identified using different sequencing methods.

Interestingly, abundances calculated for the same taxa and determined considering different hypervariable regions were quite different (Fig. 5B); this result was confirmed at different taxonomic levels. To make representation simpler, only results obtained for sample CSTR01a are reported in Fig. 5B and C, but other samples gave similar outcomes (Supplementary Table S5). This finding also evidences that analysis restricted to one specific region cannot provide an accurate estimate regarding abundance of taxonomic groups in the microbiome. Correlation between the abundance of phyla calculated using amplicons (V4 region) and those obtained with shotgun RNA, revealed that the higher correlations were observed with sequences assigned to regions V3 (R^2^ 0.83), V4 (R^2^ 0.7) and V5-V6 (R^2^ 0.89). Correlation values at lower taxonomic levels were variable; for example, at genus level the correlation calculated between “amplicons” and shotgun RNA sequences assigned to the V4 region was very low (R^2^ 0.43), while higher values were evidenced considering the V3 region (R^2^ 0.72) and the V5-V6 regions (R^2^ 0.99).

The discrepancies in abundance identified at phylum level (*Euryarchaeota*) and at genus level (*Sphaerochaeta*, *Acetivibrio*, *Peptostreptococcus* and *Methanoculleus*) were investigated in more detail considering also the sequencing method used (previous section and Fig. 4). As evidenced in Fig. 5B, “efficiency level” in taxonomic assignment of the V4 region was not identified as the main determinant for the lower abundance of *Euryarchaeota* (*Methanoculleus*) and *Spirochaetes* (*Sphaerochaeta*); in fact abundance estimated using the V4 region was close to the average value obtained with sequences mapped to other hypervariable regions. Efficiency of the V4 region was found to be more relevant for *Peptostreptococcus, Tepidimicrobium* and *Acetivibrio*. Again this was revealed by the lower abundance value obtained using V4region in comparison to the average value obtained using the other hypervariable regions. In particular for *Peptostreptococcus* and *Acetivibrio*, results were biased by an extremely high number of reads aligned on regions V9 or V5-V6 (Fig. 5).

Another finding is that for *Euryarchaeota* all the hypervariable regions (except than V3) confirmed a higher number of reads in shotgun RNA in comparison to amplicons (Fig. 5A).No clear evidences were obtained for TM7 phylum and for *Sphaerochaeta* genus.

### Identification of possible amplification biases determined by the universal primers 515 F/806 R

The presence of possible biases during PCR amplification of the 16S rRNA gene was evaluated testing limitations of universal primers 515 F/806 R to efficiently hybridize on the 16S rRNA sequences. This was performed with a two-step process based on an initial assembly of full-length 16S sequences, followed by a “virtual PCR” useful to investigate potential amplification drawbacks. Despite this approach is probably less precise than a real PCR verification, it allowed the verification of a high number of different templates in a short time. The full-length 16S rRNA sequences used for virtual PCR were obtained assembling shotgun DNA and RNA sequences with dedicated software and subsequently clustering the resulting sequences at 97% and 99% similarity level. Since universal primers used in the present study matched the V4 region, 16S rRNA obtained from the assembly were tested to verify whether their sequence included the V4 region. This procedure led to the selection of 1397 16S rRNA sequences clustered at 97% similarity and 1876 sequences clustered at 99% similarity.

Sixty-six out of 1397 bacterial sequences clustered at 97% similarity (4.7%), and 81 out of 1876 (4.3%) of those clustered at 99% similarity did not pass the “virtual amplification” (Supplementary Table S6). The percentage of failures for Archaea was higher (19%) as evidenced by the identification of 6 (out of 32) 16S rRNA sequences which failed the “virtual amplification” check. The taxonomic assignment of the sequences that failed the “virtual PCR” was investigated to verify potential drawbacks on specific taxonomic lineages. Phyla having the highest fraction of amplification failures were *Spirochaetes* (∼71%) (*Sphaerochaeta* genus), *Bacteroidetes* (∼17%) (*Thermonema* genus), *Actinobacteria* (∼16%) (*Arthrobacter* genus) and *Euryarchaeota* (∼23%) (*Methanoculleus* genus) (sequences clustered at 97% similarity). Results obtained for sequences clustered at 99% similarity were very similar. These findings suggest that the predicted fraction of 16S genes failing the amplification is limited and that, among taxa with low estimate abundance in amplicons, only *Sphaerochaeta* and possibly *Methanoculleus* were biased by failures at the PCR amplification step. Previous studies reported the presence of biases in 16S rRNA amplification for *Spirochaeta*, particularly in association with primers “63 F”, “1389 R” and “S-D-Bact-0347-a-S-19”, “S-D-Bact-0785-a-A-19”^49,50^. These findings suggest that species belonging to this phylum are particularly refractory to 16S rRNA amplicon sequencing.

The main results obtained in the present study and some suggestions to improve the taxonomic analysis of the AD microbiome were resumed in Table 1.

**Table 1:** Important remarks for analyzing the microbiome of anaerobic reactors for biogas production.

Method	16S rRNA amplicon seq.	Shotgun DNA	Shotgun RNA
Number of reads needed for accurate taxonomic analysis.	Low (>10,000). All the sequences target the 16S rRNA gene and this allows reliable investigation of the main taxa with few reads.	Very high (>1,000,000). Number of reads assigned to the 16S rRNA gene is low.	Intermediate (>100,000). Loss of reads determined by the presence of transcripts other than 16S rRNA gene is quite limited.
Possible suggestions.		Increase the number of clade-specific marker genes other than 16S rRNA using dedicated software (e.g. MetaPhlAn)	
Hypervariable regions.	Analysis targets one or two selected regions. This can reduce accuracy in calculating abundance of specific taxa (e.g. *Peptostreptococcus*, *Tepidimicrobium* and *Acetivibrio*).	Analysis targets all the hypervariable regions. This can increase both the efficiency of taxonomic analysis and the evaluation of abundance for most taxonomic groups.	Same as shotgun DNA.
Possible suggestions.	Increase the number of hypervariable regions under investigation with longer reads (e.g. using PacBio SMRT technology) or analyzing more than one amplicon. V1-V2 regions seem particularly promising to improve taxonomic results.		
Universal primers.	Universal primers introduce biases (e.g. *Sphaerochaeta* and *Methanoculleus*) due to inability of hybridizing on all the 16S rRNA molecules.	No amplification step needed, this reduces biases in taxonomic investigation.	Same as shotgun DNA.
Possible suggestions.	Perform accurate check for potential biases in 16S rRNA gene amplification. Use more than one couple of universal primers.		
Transcriptional activity.	This approach targets genomic DNA, transcriptional activity cannot be monitored and expression level of the 16S rRNA gene does not influence analysis.	Same as 16S rRNA amplicon seq.	This approach targets RNA molecules and provides insights in activity of specific taxa. Analysis can be inaccurate in determining abundance of taxa characterized by high or low activity.
Possible suggestions.	Combine different sequencing approaches to gain insights both on microbial abundance and on their activity.	Same as 16S rRNA amplicon seq.	Same as 16S rRNA amplicon seq.

### Impact of different sequencing methods on the identification of taxa influenced by oleate addition

The microbial community under investigation was sampled in two different conditions, before and after the addition of supplemental amounts (12 g/L-feed) of unsaturated fatty acids (in the form of Na-oleate) in cattle manure feedstock. In the previous sections it was demonstrated that the sequencing method had a relevant influence on the abundance of the taxa identified; for this reason, it is expected that the sequencing approach can also influence the identification of taxonomic groups changing in abundance in response to Na-oleate.

**Figure 6:**
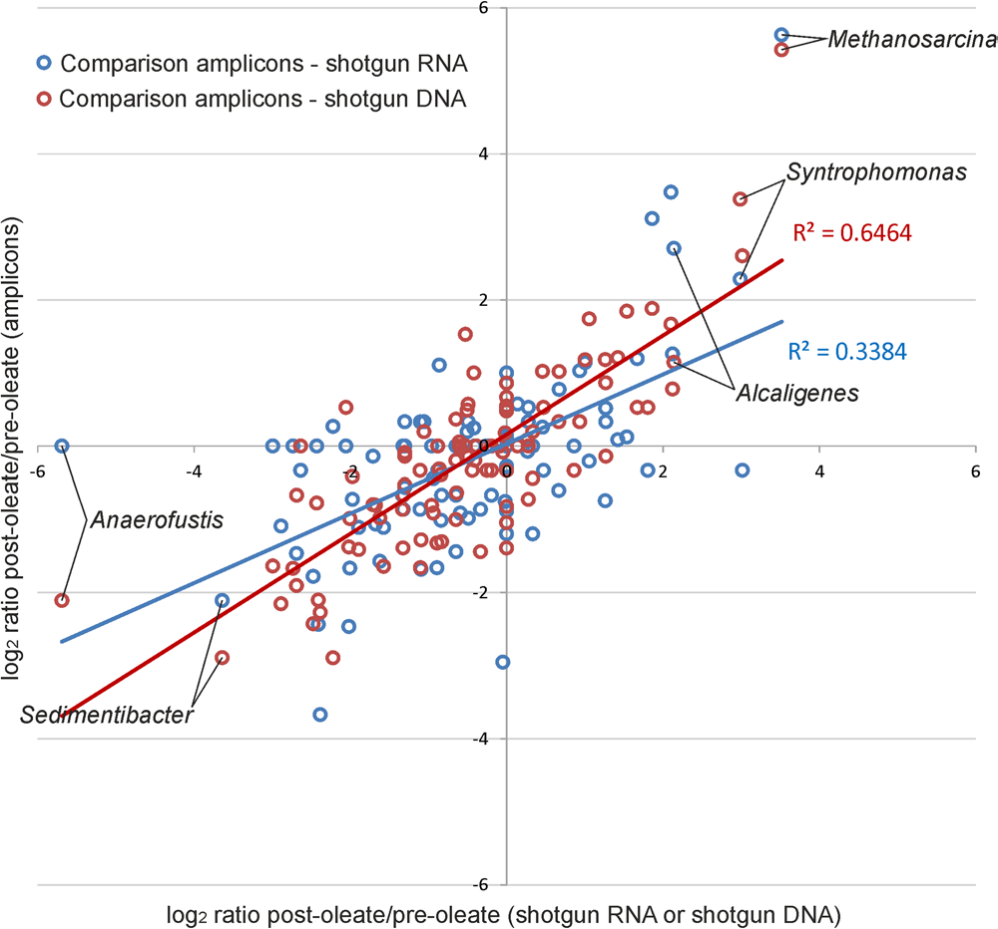
Abundance ratio (log_2_) determined for the 100 most abundant genera before and after Na-oleate addition. x and y axes report the log_2_ ratios obtained by dividing abundance level of genera “after” and “before Na-oleate” addition. “Blue dots” represent the comparison between log_2_ ratio determined for amplicon sequencing (x-axes) and log_2_ ratio determined for shotgun RNA sequencing (y-axes) (60,000 subsampled sequences). “Red dots” represent the comparison between the log_2_ ratio determined for amplicon sequencing (x-axes) and log_2_ ratio determined for shotgun DNA sequencing (y-axes).

By checking changes in abundance at genus level, results obtained from amplicon sequencing were more similar to those obtained from shotgun DNA (R^2^ 0.65) (red in Fig. 6), while they diverged from those obtained with shotgun RNA (R^2^ 0.34) (blue in Fig. 6). This is expected because in amplicon sequencing and in shotgun DNA sequencing the number of reads per OTU are mainly determined by species abundance, while in RNA sequencing abundance is influenced both by species abundance and by expression level of the 16S rRNA gene. Shotgun DNA sequencing does not depend on gene-targeted primers or PCR amplification, thus it is not affected by primer bias or chimeras and for this reason it provides a better representation of the taxonomic abundance in comparison to shotgun RNA sequencing. Despite this, analysis at the transcript level is probably more representative of the activity of microbial species^18^.

By checking results at phylum level, most of the changes in abundance determined by Na-oleate were concordant for all the three sequencing approaches (Supplementary Tables S7 and S8). Only one discrepancy was evidenced for *Spirochaetes*. In this phylum, both amplicons and shotgun DNA evidenced a decreased abundance after Na-oleate addition, 5.97 fold for amplicons (p-value 0.00127) and 4.89 fold for shotgun DNA (p-value 0.0007). Differently, results obtained from shotgun RNA revealed only a 1.97 fold decrease (p-value 0.56).

As evidenced in Fig. 6, most of the genera which underwent a modification in abundance after Na-oleate addition were confirmed by the three methods used; for example, both *Syntrophomonas* and *Alcaligenes* resulted in a markedly increased abundance, while *Sedimentibacter* was dramatically decreased. Results evidenced also some interesting inconsistences, as for example in the *Anaerofustis* genus. This taxon was not detected using shotgun RNA sequencing, while both amplicon and shotgun DNA sequencing evidenced a strong decrease after Na-oleate addition. This intriguing result suggests a very low transcriptional activity for this genus, which can be determined only using RNA-seq.

## Conclusions

This is the first study that performed an in-depth comparative evaluation of three widely used sequencing methods to investigate the taxonomic composition specifically focused on the anaerobic digestion microbiome. It was demonstrated that the classical 16S rRNA amplicon sequencing is biased by two main effects, which are the limited number of hypervariable regions investigated (V4 in the present study) and, at a less extent, the failure of universal primers to match all the 16S rRNA targets. These two biases influenced different taxonomic groups and, more specifically, amplification drawbacks were more problematic for *Euryarchaeota* and *Spirochaetes*. Interestingly, analysis of shotgun DNA reads performed using a group of clade-specific marker genes other that 16S rRNA confirms that use of this marker gene can lead to underestimation in abundance of *Euryarchaeota* in the AD system. This finding indicates also that use of multiple marker genes, or analysis at transcriptional level, could improve the evaluation of abundance for crucial taxonomic groups. Moreover, it is concluded that the absolute abundance level of different taxa is markedly influenced by the selected hypervariable region and also by the set of sequences used to train the Bayesian classifier. These two limitations suggest caution in considering absolute abundance levels of taxa determined using amplicon sequencing results. It was also evidenced that investigation of more than one hypervariable region (including for example V1 and/or V2) can improve the quality of the results. From a general point of view, the abundance estimation obtained using 16S rRNA amplicons is well correlated with the corresponding one obtained using shotgun DNA sequencing, while more diverse results were found in the comparison with the shotgun RNA data.

## Methods

### Configuration of the biogas reactors, management and samples collection

Shotgun DNA and shotgun RNA sequences analyzed in this study were obtained from previous studies^32,33^, while 16S amplicons were specifically generated and sequenced for this comparative analysis. A detailed description of the reactors used and operational conditions was previously reported^32^. Experiments were carried out in triplicate continuous stirred tank reactors (CSTR), denoted as CSTR01, CSTR02, and CSTR03 having a 1.5 L working volume. All reactors were equipped with magnetic stirrers and thermal jackets were used to maintain the operating temperature at 54 ± 1 °C. Initially, the reactors were inoculated with thermophilic inoculum obtained from Snertinge biogas plant, Denmark. During the first period the reactors were fed exclusively with cattle manure and then the influent feedstock was supplemented with sodium oleate (12 g/L-feed). The hydraulic retention time (HRT) of all reactors was kept constant at 15 days. Samples for genomic and RNA extraction (∼15 mL each) were collected from each reactor during the steady state condition of each period (i.e., period with stable biogas production with a daily variation lower than 10% for at least 5 days). The three samples (biological replicates) obtained from the first period were indicated as CSTR01a, CSTR02a, and CSTR03a, while the samples obtained from the second period were indicated as CSTR01b, CSTR02b, and CSTR03b.

### DNA/RNA extraction, shotgun DNA, shotgun RNA and amplicon sequencing

Barley residues present in the manure were removed using a 100 μm nylon cell strainer filter as previously described^29^. The filtered samples were centrifuged at 5000 rpm for 10 min and the supernatant was discarded leaving ∼2 g of material. To avoid RNA degradation, 3.5 mL of phenol/chloroform (pH 6.7/8.0) were mixed with isoamyl alcohol (25:24:1) (Amresco, Incorporated) and were added to the pellet after centrifugation. The samples were immediately processed for extraction of nucleic acids. Total RNA was extracted from 2 g of pellet using the RNA PowerSoil Kit (MO BIO laboratories, Carlsbad, CA). Genomic DNA was extracted from the same samples after separation from RNA, using the RNA PowerSoil® DNA Elution Accessory Kit (MO BIO laboratories, Carlsbad, CA). The quality and the quantity of the nucleic acids were determined both using NanoDrop (ThermoFisher Scientific, Waltham, MA) and Qubit fluorometer (Life Technologies, Carlsbad, CA). RNA integrity was determined with Agilent Bioanalyzer, genomic DNA integrity was determined using agarose gel electrophoresis and results were previously reported^32,33^. RNA libraries were prepared using the TruSeq RNA Library Preparation Kit (Illumina, San Diego, CA), while genomic libraries were prepared with Nextera DNA Library Preparation Kit (Illumina, San Diego, CA, USA). The V4 region of the 16S rRNA gene was amplified using universal primers 515F-806R from the same genomic samples used for shotgun sequencing^4^. The libraries obtained from RNA samples and from amplicons were paired-end sequenced (2 × 150 bp) using MiSeq system (Illumina, San Diego, CA). Shotgun RNA sequencing generated in the six samples 2206946, 2317236, 2440269, 2724922, 2688495 and 2307647 paired-end sequences mapped on the 16S rRNA gene; amplicons generated 928909, 1453725, 2123513, 1718869, 1533612 and 1814367 paired-end sequences. Genomic DNA libraries prepared with the Nextera kit were paired-end sequenced (2 × 150 bp) using one lane of the Illumina HiSeq 2500 (Illumina, San Diego, CA, USA) (~250 million filtered reads) and resulted in 82580, 96738, 82278, 121755, 107002 and 99861 paired-end sequences mapped on 16S rRNA sequences (from 0.35% to 0.51% of the total reads depending on sample).

### Bioinformatics analysis

Reads in FASTQ format were quality-filtered (leading:20, trailing:20, slidingwindow:4:20, minlen:100) and adaptors were removed using Trimmomatic software (v0.33)^51^. Shotgun reads matching the 16S rRNA gene were selected using riboPicker (standalone-0.4.3)^52^ after reads alignment on SILVA and Ribosomal Database Project (RDP) databases. Paired-end reads were merged using FLASH (v1.2.11)^53^ using options (−M 80) for amplicons and (−M 100 − x 0.01) for shotgun reads. Conversion of reads from fastq to fasta format was performed with QIIME (1.9.0 + dfsg-0biolinux5) “convert_fastaqual_fastq.py”^6^. Chimera sequences were removed using usearch (7.0.1090_i86linux32) (-uchime option) and Greengenes as reference database. Taxonomic assignment was performed using Bayesian RDP classifier^7^ trained with RDP (v11)^9^, Greengenes (13 08), or SILVA (v128)^8^. Output of RDP classifier was further analyzed using self-written perl scripts to accelerate the examination of results at different taxonomic levels. Sequences were taxonomic assigned using Bayesian classifier without a preliminary clustering-based step. This choice was determined by the random distribution of the shotgun DNA and RNA sequences on the 16S rRNA gene, a characteristic which made the OTU-based approach impractical^12^. To overcome this limitation, Illumina reads were directly assigned to the taxonomy after removal of the large number of “non-16S sequences” present in the shotgun DNA and RNA samples. This “pre-filtering step”, performed with riboPicker software^52^, selected only sequences aligned on the 16S rRNA gene and made more robust evaluation of the taxonomic results. In the comparison between taxonomic results independently obtained for PE (For and Rev) sequences, taxonomic assignment has been performed using RDP classifier (trained of Greengenes) and results were compared using self-written perl scripts. Briefly, RDP results obtained for the two paired-ends were compared and the “lowest” concordant taxonomic assignment was selected. The script calculated percentage of concordant results for each taxonomic level and reported as output a file with the same format than RDP classifier software.

Analysis of the minimum number of reads providing a reliable taxonomic result was performed starting from RDP classifier output (trained on Greengenes DB) and using self-written perl scripts to calculate the taxonomic results on a subset of randomly chosen sequences (perl “rand” function). The script allows the selection of parameters such as “repeat random resampling N times” (selected N = 5 resampling) and “increase of K the number of reads at each step” (selected K = 1000 between 0 and 10000, selected K = 10000 between 10000 and 100000, selected K = 100,000 between 100,000 and 700,000).

Assembly of shotgun reads assigned to the 16S rRNA gene was performed using EMIRGE^54^. Sequences were clustered at 97% and 99% similarity using QIIME (1.9.0 + dfsg-0biolinux5) “pick_otus.py” software^6^ and taxonomy of the 16S rRNA sequences obtained were assigned using RDP classifier trained on Greengenes database. Presence on the 16S rRNA sequences of the V4 region was verified by aligning the 16S rRNA sequence with nhmmer (v3.1b1) (parameter -E 0.0001) on two hidden markov models (bac.ssu.rnammer.hmm, bac.ssu.rnammer.hmm) obtained from RNAmmer (v1.2) software^55^. Sequences including the V4 region were recovered by considering the start/end positions of the alignment on hidden markov models. A “virtual PCR” was performed using MFEprimer-2.0^56^ in order to verify the ability of universal primers to match each sequence. MFEprimer-2.0 software was launched on each 16S rRNA sequence using an automated pipeline which also parsed and verified the output files obtained from the analysis by selecting the sequences which passed the virtual amplification test. For each taxon, the number of sequences failing the virtual amplification test was compared with the total number of sequences identified for the same taxon.

Analysis of the shotgun DNA sequences based on ∼1 million marker specific genes was performed aligning the reads with Bowtie2 (v2.2.4)^57^ and analyzing results obtained with MetaPhlAn 2 software (2.2.0)^47^.

### Data availability statement

Shotgun DNA data were submitted to SRA database under project PRJNA283298, SRP058179) and using the following accession numbers: CSTR01b (SRX1023882), CSTR02b (SRX1023884), CSTR03b (SRX1023885), CSTR01a (SRX1022794), CSTR02a (SRX1023859), CSTR03a (SRX1023867). Shotgun RNA data were associated to the same project and with the following accession numbers: CSTR01a (SRX1535498), CSTR02a (SRX1535533), CSTR03a (SRX1535534), CSTR01b (SRX1535507), CSTR02b (SRX1535535), CSTR03b (SRX1535536). Amplicons were associated to the same project using the following accession numbers: CSTR01a (SRX3011210), CSTR02a (SRX3013414), CSTR03a (SRX3013415), CSTR01b (SRX3013420), CSTR02b (SRX3013427), CSTR03b (SRX3013442).

## Electronic supplementary material

Supplementary Table S1

Supplementary Table S2

Supplementary Table S3

Supplementary Table S4

Supplementary Dataset S1

Supplementary Table S5

Supplementary Table S6

Supplementary Table S7

Supplementary Table S8
