# Supplementary material for: Taxonomy of anaerobic digestion microbiome reveals biases associated with the applied high throughput sequencing strategies
Source: Sci Rep. 2018 Jan 31;8:1926. doi: 10.1038/s41598-018-20414-0 (PMC5792648; doi:10.1038/s41598-018-20414-0)
Supplement: Supplementary file 5 — Supplementary Dataset S1 [file 41598_2018_20414_MOESM5_ESM.doc]

**Supplementary Information**

**Taxonomy of anaerobic digestion microbiome reveals biases associated with the applied high throughput sequencing strategies**

Stefano Campanaro ^1^, Laura Treu ^2,*^, Panagiotis G. Kougias ^2^, Xinyu Zhu ^2^ and Irini Angelidaki ^2^

^1^ Department of Biology, University of Padova, Via U. Bassi 58/b, 35121, Padova Italy

^2^ Department of Environmental Engineering, Technical University of Denmark, 2800 Kgs. Lyngby, Denmark

* Corresponding author at: Department of Environmental Engineering, Technical University of Denmark, Bygningstorvet Bld 115, 2800 Lyngby, Denmark. E-mail address: latr@env.dtu.dk (L. Treu).

**Supplementary Dataset S1**

***Comparison between taxonomic results obtained merging forward and reverse paired-ends and those independently obtained on forward and reverse***

Forward and reverse reads can be merged with dedicated software (FLASH in the present study) in order to obtain longer sequences which can provide more reliable taxonomic assignments (merged ends analysis). This process can be performed more efficiently for amplicons due to the defined distance of the pairs, while it is trickier for random shotgun libraries because the size of the DNA molecules spans over a wider range than amplicons. In the present study the improved efficiency in taxonomic assignment obtained using the “merged ends” analysis was measured. Forward and reverse pairs of each read were independently classified using Bayesian classifier (separate ends analysis) and results were subsequently compared. Only results that were in agreement were reported as output (see Materials and Methods section for more details). The number of taxonomic assigned reads was higher using the merged sequences (~15% more at phylum level). The lower number of taxonomic assigned reads in the “separate ends” analysis was mainly due to lower quality of the reverse reads. This confirms previous findings by Jeraldo and colleagues (Jeral*do et a*l., 2014). A comparison between abundance determined at genus level using “merged ends” and “separate ends” evidenced a very high correlation coefficient (R^2^ > 0.99) (Figure S1) suggesting that both methods can provide a reliable taxonomic result. This analysis suggests that, when possible, PE reads should be merged, but the influence of this process on the taxonomy profile obtained was limited.

Jeraldo, P., Kalari, K., Chen, X., Bhavsar, J., Mangalam, A., White, B. *et al.* (2014) IM-TORNADO: a tool for comparison of 16S reads from paired-end libraries. *PLoS One* **9**: e114804.


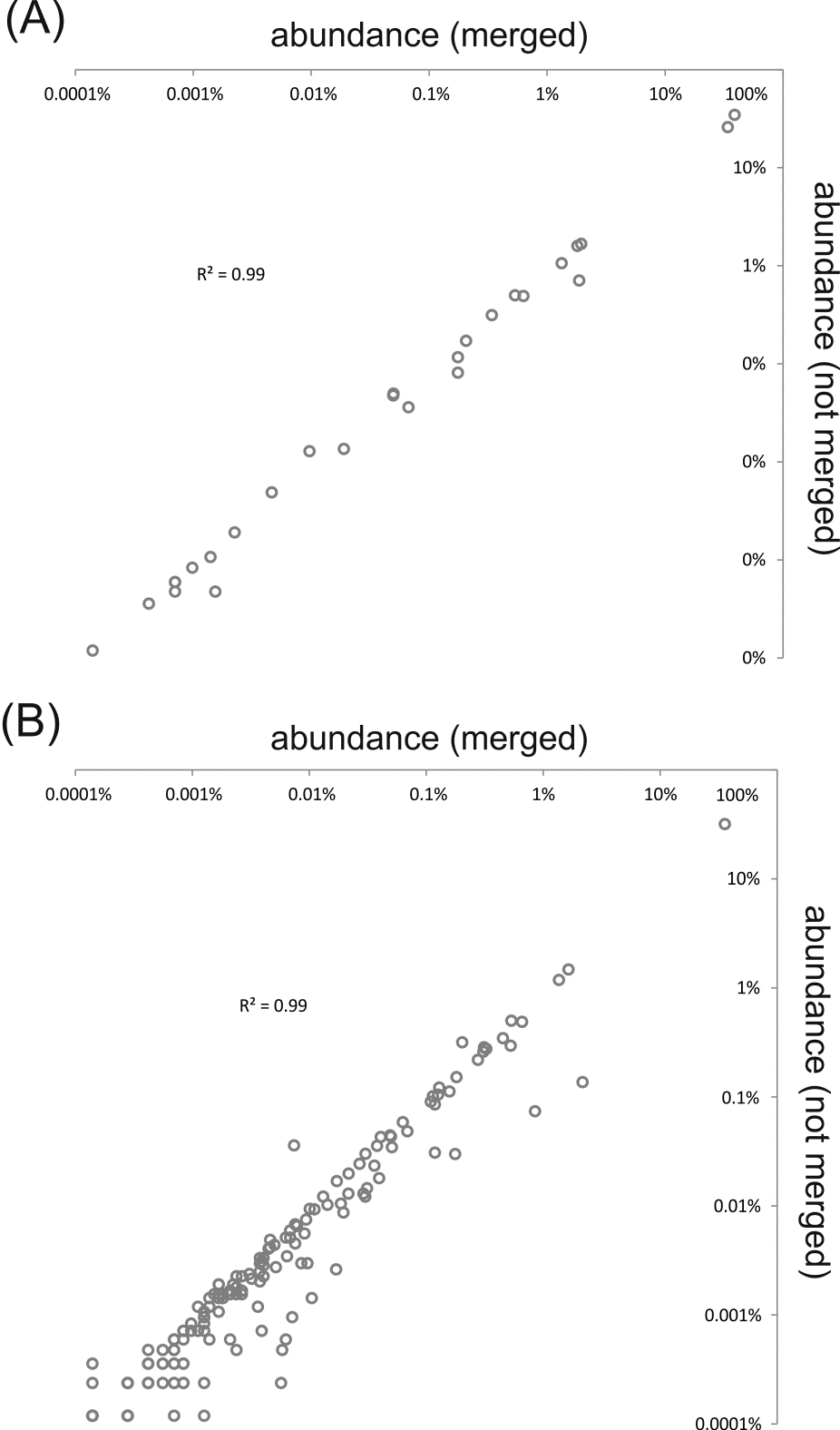


**Fig. S1.** Comparison of taxonomic assignment obtained from merged paired-end and unmerged paired-end reads. Abundance determined for different phyla (A) and genera (B) calculated as fraction of reads on the total number of reads for the sample CSTR01a. Correlation of the abundance values is reported as R^2^. For most of the taxa the fraction of reads assigned is slightly lower assigning taxonomy independently to the forward and reverse reads (y axes) then using the merged reads (x axes).
